# Identification of Serum miRNAs as Effective Diagnostic Biomarkers for Distinguishing Primary Central Nervous System Lymphoma from Glioma

**DOI:** 10.1155/2022/5052609

**Published:** 2022-04-20

**Authors:** Pei-pei Si, Xiao-hui Zhou, Zhen-zhen Qu

**Affiliations:** ^1^Department of Neurology, Key Laboratory of Neurology of Hebei Province, The Second Hospital of Hebei Medical University, Shijiazhuang, Hebei, China; ^2^Department of Anesthesiology, The First Hospital of Hebei Medical University, Shijiazhuang, Hebei, China

## Abstract

Invasive surgical cerebrum biopsy results in delayed treatment for the definitive diagnosis of primary central nervous system lymphoma (PCNSL). The existent research was aimed at confirming the underlying diagnostic miRNAs of distinguishing PCNSL from glioma. A publicly available miRNA expression profiles (GSE139031) from adult PCNSL as well as glioma specimens were provided by GEO datasets. Differentially expressed miRNAs (DEMs) were filtered between 42 PCNSL patients and 170 glioma patients. Candidate miRNAs were identified through SVM-RFE analysis and LASSO model. ROC assays were operated to determine the diagnostic value of serum miRNAs in distinguishing PCNSL from glioma. StarBase v2.0 was applied to screen the targeting genes of miRNAs, and KEGG analysis was applied using the targeting genes of miRNAs. In this study, we identified 12 dysregulated miRNAs between PCNSL and glioma samples. The ten critical miRNAs (miR-6820-3p, miR-6803-3p, miR-30a-3p, miR-4751, miR-3918, miR-146a-3p, miR-548am-3p, miR-371a-3p, miR-487a-3p, and miR-4756-5p) between these two algorithms were ultimately identified. The results of KEGG revealed that the targeting genes of hsa-miR-3918 were primarily related to MAPK signal pathway, PI3K-Akt signal pathway, and human papillomavirus infection. Overall, bioinformatics analysis revealed that ten miRNAs are potential biomarker for distinguishing PCNSL from glioma.

## 1. Introduction

Primary central nervous system lymphoma (PCNSL) and glioma are both highly malignant tumors of the central nervous system; however, different treatment procedures were applied for the above two tumors [1, 2]. In individuals with gliomas, surgery is suggested in order to improve symptoms and avoid recurrence, but it has little effect on survivals of PCNSL [3]. Glioma and PCNSL are rapidly progressing diseases associated with poor prognosis [4, 5]. As these tumors share similar clinical and radiological characteristics, it might be difficult to distinguish them from one another [6, 7]. Diagnostic and therapeutic techniques involving surgery are necessary in some situations. Thus, it is critical to identify fresh sensitive biomarkers for distinguishing PCNSL from glioma in order to provide patients with appropriate treatment.

MicroRNAs (miRNAs), which is a kind of small, highly conserved, and noncoding RNAs, can straightway bind to certain sequence-specific positions of target genes' 3′-UTRs (3′ untranslated regions) to inhibit the expression of these genes [8, 9]. They widely take part in many biological processes such as cell communication, development, and differentiations [10, 11]. During the past decades, miRNAs have been implicated in the growth and metastasis of tumors in the past by functioning as tumor oncogenes or tumor suppressors, according to the recent research [12, 13]. miRNAs that are present in the blood or tumors can be useful biomarkers and tumor suppressors in the case of brain cancers [14, 15]. However, the underlying miRNAs as new diagnostic biomarkers of distinguishing PCNSL from glioma have not been investigated.

In the paper, we downloaded two microarray datasets of PCNSL and glioma from the GEO datasets. Differentially expressed miRNA (DEM) assays were performed between the PCNSL and glioma. Machine learning algorithms were applied to screen and confirm diagnostic biomarkers in PCNSL and glioma. From this study, we suspect that serum miRNAs can be required to precise distinct lots of PCNSL patients from glioma patients.

## 2. Materials and Methods

### 2.1. Data Resource and Preprocessing

miRNA expression profiles of glioma and PCNSL were found out in publicly gettable GEO in NCBI (http://www.ncbi.nlm.nih.gov/geo/). Inclusion standards were human miRNA expression data, glioma samples and PCNSL samples, and total count of samples ≥ 50. At last, GSE139031 was downloaded from NCBI GEO, including 42 PCNSL patients and 170 glioma patients.

### 2.2. Identification of DEMs

Statistical software R (version 3.3.2, https://www.r-project.org/) and packages of Bioconductor (http://www.bioconductor.org/) were used for significance analysis of DEMs between PCNSL specimens and glioma specimens. The limma package of R (http://www.bioconductor.org/) was applied to count the *P* value and FDR, separately. The DEMs were filtered out on the basis of adjusted *P* value < 0.05 as well as ∣ logFC  | ≥1.2.

### 2.3. Candidate Diagnostic Biomarker Screening

Two machine learning algorithms were applied to forecast disease situation in order to identify important prognostic variables. The least absolute shrinkage and selection operator (LASSO) can increase prediction accuracy using regularization [16]. LASSO regression algorithm was implemented with the “glmnet” package in R to confirm the miRNAs remarkably related to the difference of PCNSL and glioma specimens. Support vector machine (SVM) can be widely used in classification and regression as a supervised machine learning technology [17]. RFE algorithm was applied to choose the best miRNAs of the cohort in order to avoid overfitting. SVM-RFE was used to choose proper characteristics in order to find out the miRNAs of the highest discriminating power. Overlapping miRNAs between the above two algorithms were identified as critical diagnostic biomarkers.

### 2.4. Diagnostic Values of Serum miRNAs in Distinguishing PCNSL from Glioma

To examine the predicted values of the confirmed markers, we produced an ROC curve with miRNA expression data of PCNSL as well as glioma specimens. The region under the ROC curve (AUC) value was used to find out the diagnostic effectiveness in screening PCNSL from glioma specimens.

### 2.5. GO and KEGG Pathway Enrichment Analyses of the Targeting Genes of miRNAs

StarBase v2.0 was applied to screen the targeting genes of miRNAs [18]. GO analysis contains three types: molecular function, biological process, and cellular component. GO analysis was operated using gseGO function in clusterProfiler package. The adjusted *P* value < 0.05 was as the cutoff standard. The GOenrich software used a network diagram to show the relationships between the most important GO terms and the genes involved. Besides, KEGG pathway enrichment analysis was performed using gseKEGG functions in clusterProfiler package. Accordingly, the cutoff level for the adjusted *P* value was set at 0.05.

### 2.6. Statistical Analysis

All statistical analyses were conducted using R software 3.5.3. Statistical significance was given at a possible value of *P* < 0.05. ROC curves were applied to determine the predicted accuracy of dysregulated miRNAs. Differences between groups were compared by the Wilcox test through R software. “Glmnet” was used to conduct LASSO regression analysis, while the e1071 package was used to run the SVM algorithm in R.

## 3. Results

### 3.1. Identification of DEMs between Glioma and PCNSL Samples

**Figure 1:**
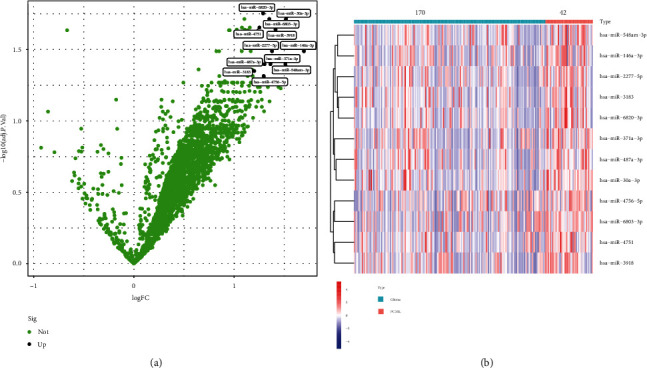
Volcano plot (a) and heat map (b) showed the differentially expressed miRNAs between PCNSL and glioma specimens.

We used limma package to screen DEMs between glioma and PCNSL samples. As shown in Figures 1(a) and 1(b), we identified 12 upregulated miRNAs in PCNSL samples, including miR-6820-3p, miR-6803-3p, miR-30a-3p, miR-4751, miR-3918, miR-2277-5p, miR-146a-3p, miR-548a-3p, miR-371a-3p, miR-487a-3p, miR-3183, and miR-4756-5p.

### 3.2. Identification of the Diagnostic miRNAs

**Figure 2:**
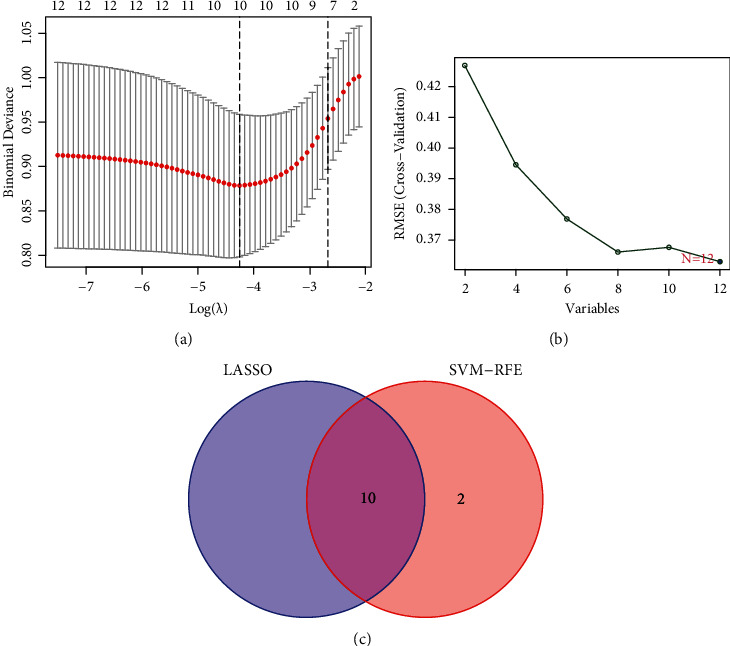
Filtering course of diagnostic candidates of distinguishing PCNSL from glioma. (a) Tuning characteristic option in LASSO model. (b) A plot of biomarkers option through SVM-RFE algorithm. (c) Venn diagram illustrating 4 diagnostic markers shared by LASSO and SVM-RFE algorithms.

Two distinct algorithms were applied to filter underlying markers. The DEMs were decreased with LASSO regression algorithm, leading to the confirmation of 10 miRNAs as diagnostic biomarkers for distinguishing PCNSL from glioma (Figure 2(a)). A series of 5 characteristics among the DEMs were decided with the SVM-RFE algorithm (Figure 2(b)). 10 overlapping miRNAs (miR-6820-3p, miR-6803-3p, miR-30a-3p, miR-4751, miR-3918, miR-146a-3p, miR-548am-3p, miR-371a-3p, miR-487a-3p, and miR-4756-5p) between the 2 algorithms were chosen finally (Figure 2(c)).

### 3.3. The Expression and Diagnostic Value of Ten miRNAs in Distinguishing PCNSL from Glioma

**Figure 3:**
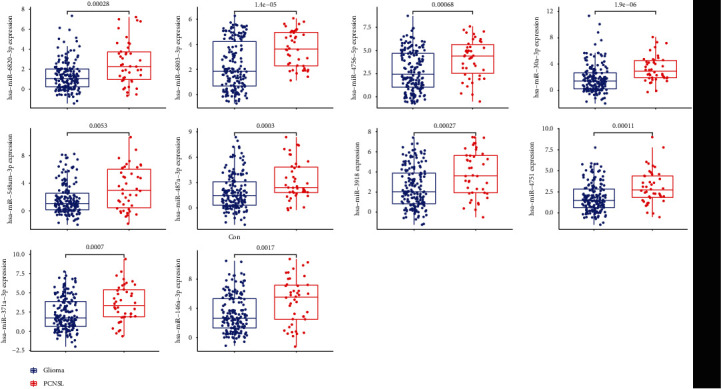
The expression pattern of miR-6820-3p, miR-6803-3p, miR-30a-3p, miR-4751, miR-3918, miR-146a-3p, miR-548am-3p, miR-371a-3p, hsa-miR-487a-3p, and hsa-miR-4756-5p in PCNSL and glioma samples.

**Figure 4:**
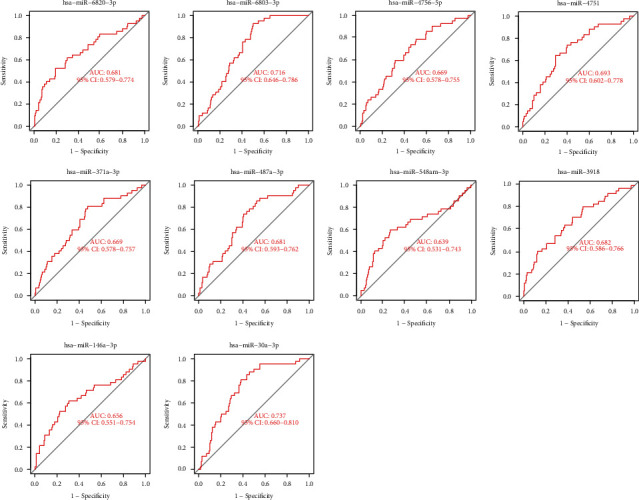
The ROC curve of diagnostic effectiveness of then diagnostic markers, including miR-6820-3p, miR-6803-3p, miR-30a-3p, hsa-miR-4751, miR-3918, miR-146a-3p, miR-548am-3p, miR-371a-3p, miR-487a-3p, and miR-4756-5p.

The expression pattern of the above ten miRNAs is shown in Figure 3. In addition, we performed ROC assays to determine the diagnostic value of ten miRNAs using GSE139031. As shown in Figure 4, all ten miRNAs showed a powerful discrimination ability.

### 3.4. GO and KEGG Assays Based on the Targeting Genes of hsa-miR-3918

**Figure 5:**
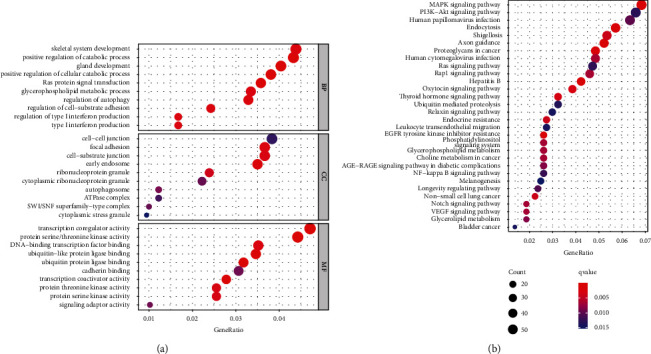
Functional analysis based on the targeting genes of hsa-miR-3918. (a) GO enrichment was shown on a bubble graph. (b) KEGG assay was shown on a bubble graph.

StarBase v2.0 was applied to screen the targeting genes of miRNAs, and 3787 genes were identified. GO assays revealed that the targeting genes of hsa-miR-3918 were mainly enriched in skeletal system development, positive regulation of catabolic process, cell-cell junction, cell-substrate junction, transcription coregulator activity, and protein serine/threonine kinase activity(Figure 5(a)). The results of KEGG revealed that the targeting genes of hsa-miR-3918 were primarily related to MAPK signal pathway, PI3K-Akt signal pathway, and human papillomavirus infection (Figure 5(b)).

## 4. Discussion

PCNSL is a rare, aggressive brain neoplasm that accounts for roughly 2-6% of primary brain tumors [19]. In comparison, GBM is the most common and serious glioma subtype, in the proportion of almost 50% of dispersed gliomas [20]. The strategies on how to treat glioma and PCNSL is different substantially [21, 22]. As to glioma, the present therapeutic is maximum tumor resection and radiation therapy and chemotherapy with temozolomide afterwards [23]. However, for PCNSL, methotrexate-based chemotherapy is a common method after stereotactic intracranial biopsy. So, preoperative distinction of glioma and PCNSL is of high clinical relation. In present days, more and more researches have illustrated the dysregulation of miRNAs in various tumors [24, 25]. In addition, the prognostic and diagnostic values of miRNAs in many types of tumors have been frequently reported [26, 27]. However, whether miRNAs can be used as novel biomarkers for distinguishing PCNSL from glioma has not been investigated.

In this study, we analyzed GEO datasets and screened 12 dysregulated serum miRNAs between PCNSL patients and glioma patients. Interestingly, the 12 miRNAs are all upregulated miRNAs in PCNSL patients. According to 2 machine learning algorithms, ten diagnostic markers were confirmed, including miR-6820-3p, miR-6803-3p, miR-30a-3p, miR-4751, hsa-miR-3918, miR-146a-3p, miR-548am-3p, miR-371a-3p, miR-487a-3p, and miR-4756-5p. Previously, the function of the above miRNAs in tumor progression has been reported. For instance, Wang et al. indicated that miR-30a-5p was highly expressed in glioma and its silence inhibited the transformation of glioma cells via regulating NCAM [28]. Han and Wang found that miR-3918 expressions were distinctly downregulated in glioma and its overexpression suppressed the proliferation and invasion of glioma cells via decreasing EGFR to modulate PI3K/AKT signal [29]. However, the effects of miRNAs in PCNSL were rarely reported. Our findings may provide a new clue for other researchers to further explore whether the above ten biomarkers may influence the progression of PCNSL.

Numerous studies have suggested that some miRNAs are aberrantly expressed in tumors and involved in the development and progression of various tumors via targeting tumor-related proteins, such as P53 and ROCK1 [30, 31]. To further explore the possible function of miR-3918, we screened the possible targeting genes of miR-3918 by the use of StarBase 2.0. Finally, 3787 genes were identified. Then, we performed KEGG assays and found that the above genes were mainly enriched in several tumor-related pathways, such as MAPK signal pathway, PI3K-Akt signal pathway, and Ras signal pathway, suggesting that miR-3918 may be involved in the regulation of these pathways. Importantly, miR-3918 has been reported to modulate ERK and PI3K/AKT signals in glioma, which was consistent with our findings [29]. However, its potential function in glioma remained largely unclear. More experiments were needed.

In the present research, the quality of these data cannot be ensured because the gene expression results applied for complex analysis were from various institutions and accessed from available databases in public. Then, our findings should be further demonstrated by additional findings from biological experiments and large-scale multicenter clinical researches because our results were from the comprehensive in silico study.

## 5. Conclusions

We firstly reported that ten serum miRNAs can serve as novel diagnostic biomarkers for distinguishing PCNSL from glioma. Our findings may provide new insights for future studies on the occurrence and progression of PCNSL and glioma.

## Data Availability

The data used to support the findings of this study are available from the corresponding author upon request.
